# “I Am Very Happy That We Are Such Beautiful People”: Lived Experiences, Perceived Discrimination, and Mental Health in an LGBTIQ+ Community in Turkey

**DOI:** 10.1002/jcop.70087

**Published:** 2026-02-06

**Authors:** Buket Kara, Defne Güzel, Semih Özkarakaş, Doğa Eroğlu‐Şah, Umut Şah

**Affiliations:** ^1^ Division of Health Research Lancaster University Lancaster UK; ^2^ Özgür Renkler Derneği [Free Colours Association] Bursa Turkey; ^3^ European University of Lefke Mersin Turkey

**Keywords:** co‐production, community‐based research, LGBTIQ+, mental health, perceived discrimination, resilience, sexual and gender minority

## Abstract

Co‐produced by LGBTIQ+ activists and academic researchers, this study gave voice to an understudied LGBTIQ+ community in Turkey to narrate their lived experiences and examined their exposure to discrimination in various areas of their lives in relation to their mental health. The study utilized a mixed‐method design, where 61 individuals who identified as LGBTIQ+, aged 18–47, responded to an online survey. The quantitative tools included questionnaires assessing mental well‐being, psychological symptoms, resilience, and perceived discrimination. Qualitatively, participants responded to open‐ended questions regarding their lived experiences, such as coming out, access to healthcare, and self‐care practices. Participants were frequently exposed to various forms of discrimination, which were associated with lower mental well‐being and higher psychological symptoms. However, personal resilience factors lowered or diminished the negative role of discrimination on mental health. Identity‐based lived experiences and practices further provided an in‐depth picture of life of LGBTIQ+ individuals in this community and how they overcome adversity.

## 1 Introduction

Individuals who identify as LGBTIQ+ (lesbian, gay, bisexual, trans, intersex, and other sexual orientations and gender identities) in Turkey have been exposed to social exclusion, violence, and discrimination in every part of their lives (Cakmak 2022; Yildirim 2024), a pattern observed worldwide. Such experiences constitute an obstacle for their social integration, as well as economic and non‐economic involvement in social life in Turkey (Guzel 2017). Due to the rise of human rights‐oriented work, the formation of LGBTIQ+ self‐organizations, and the increase in the visibility of LGBTIQ+ subjects, there is an increase in academic studies and fieldwork focusing on the welfare and rights of LGBTIQ+ people worldwide (Poteat et al. 2021). Despite this, the violence, discrimination, and exclusion practices against LGBTIQ+ people continue.

Investigating the lived experiences and discrimination of LGBTIQ+ people holds great potential to inform practice and policies to promote well‐being and resilience in LGBTIQ+ communities. With this aim, the current study was designed and implemented by a local LGBTIQ+ organization to explore the relations between lived experiences, discrimination, and mental health in their community living in Bursa, Turkey.

### 1.1 Discrimination Against LGBTIQ+ People in Turkey

In Turkey's heteronormative and cisnormative social order, LGBTIQ+ people frequently encounter discrimination, violence, and otherization in many aspects of their lives due to their sexual orientation and gender identity (SOGI). Being exposed to humiliation, physical violence, and social exclusion in addition to the discrimination at home, at school and work, in public and private spaces are examples of these encounters (Cakmak 2022; Guzel 2017; Karaca 2019; Karakas 2018; Ozer 2019; Senel 2014). The fear of being subjected to violence and/or disowned by the family or friends prevents LGBTIQ+ people from coming out to their loved ones (Goregenli 2009). Social exclusion and discrimination have also an impact on LGBTIQ+ people's educational practices and legal rights, forcing them to drop out or change schools (Lambdaistanbul 2010; Yilmaz and Gocmen 2015). At work, the discrimination based on SOGI starts with the recruitment processes and exclusion from the employment area, and continues with the practices of forced resignation, firing, humiliation, and sexual harassment (Kaos 2019a; 2019b). Other examples of discrimination are reported as underpayment, delay/prevention of promotion, prevention of exercising rights, and being graded down in performance evaluations (Beyaz 2018).

Media further increases and normalizes the violence and discrimination in society against LGBTIQ+ people (Guzel 2017). For example, the assaults against LGBTIQ+ individuals might be articulated as the victim's fault instead of the perpetrator's or might be totally ignored and overlooked (Ozer 2019). An analysis of 2442 news, columns, and interviews published in 2018 in print media in Turkey revealed that half of these pieces embraced hate speech and discriminatory discourses, criminalized LGBTIQ+ people, and incited violence against them (Kaos 2018b). Considering the significant influence of the media discourses on the public, these statements are substantially important in terms of LGBTIQ+ representation.

SOGI are not defined in the constitution of Turkey, which leaves LGBTIQ+ individuals unprotected in the face of discrimination and violence (Kaos 2019c). Indeed, Turkey is identified as the second‐worst country in Europe in terms of LGBTIQ+ equality laws and policies (the Rainbow Index; ILGA Europe 2020). Hence, the obstacles in the way of access to law and justice prevent LGBTIQ+ people from relying on laws and/or taking legal actions to protect their rights (Yilmaz and Gocmen 2015). Furthermore, Turkey withdrew from the Istanbul Convention in 2021, a landmark treaty aimed at preventing violence against women and domestic abuse. Although not explicitly about LGBTIQ+ rights, government officials justified the withdrawal by claiming the convention was being used to promote LGBTIQ+ identities, reinforcing systemic discrimination (Amnesty International 2021).

### 1.2 Psychological Consequences of Discrimination

Individuals who identify as LGBTIQ+ often experience minority stress, which refers to the chronic and structural stressors expressed by individuals from marginalized groups due to their minority status (Meyer 2003). These stressors can arise from prejudice, societal stigma, exclusion, discrimination, and lack of social support, leading to negative impacts on mental and physical health (Douglass and Conlin 2020; Plöderl and Tremblay 2015), as well as barriers to economic and educational opportunities (Hatzenbuehler 2009) and access to healthcare (Mayer et al. 2008). LGBTIQ+ people in Turkey experience anxiety and fear because of the negative experiences they had during their coming out processes (Kabacaoglu 2015), which may further lead to suicidal ideation, self‐injuries, and psychological symptoms in LGBTIQ+ people to a greater extent in comparison to the general society (Mustanski et al. 2010; Watson and Tatnell 2022). Notably, a few rare studies conducted in Turkey with trans communities indicated that the prevalence of lifelong psychological disorders was around 60% (Basar and Oz 2016), suicide ideation was as high as 50% (Ordek 2014), and suicide attempts were around 35% (Lambdaistanbul 2010; Ordek 2014).

Several psychosocial factors play a role in buffering the negative effects of minority stress to protect LGBTIQ+ people and promote their resilience. Resilience is defined as the capacity of individuals to adapt successfully in spite of adversity (Masten 2014), explaining how individuals overcome adverse experiences and various difficulties such as poverty, discrimination, and traumatic experiences, from childhood to advanced ages. Personal resilience factors such as emotional regulation, self‐awareness, and coping strategies play a critical role in mitigating the effects of discrimination. For example, research shows that hope, courage, and optimism are key traits associated with resilience in LGBTIQ+ populations, helping individuals maintain a positive outlook despite adversity and are linked to better mental health outcomes (Atalay 2019; Kwon and Hugelshofer 2010; Peel et al. 2022).

Relational resilience is equally important. Social support from friends and family has been consistently identified as a protective factor for mental health outcomes among LGBTIQ+ people. Studies show that maintaining close connections with supportive individuals reduces the impact of discrimination and also enhances overall well‐being (Moran et al. 2018; Peel et al. 2022). In Turkey, where societal stigma often isolates LGBTIQ+ individuals, relational resilience through peer networks or support groups becomes particularly vital. A limited number of studies focused on the strengths and resilience of the LGBTIQ+ people in Turkey, showing that factors such as a socially supportive environment, social activism, and friends' support promoted resilience in transgender people and enabled them in their transition process to cope with stress (Basar and Oz 2016). Similarly, perceived social support was found to be protective against stress in LGBTIQ+ individuals in Turkey (Atalay 2019).

Coping strategies are central to fostering both personal and relational resilience among LGBTIQ+ individuals, particularly in the face of minority stress. These strategies can be approach‐oriented methods, such as seeking social support or problem‐solving, or avoidance‐oriented methods, such as denial or escape (Compas et al. 2001). Notably, studies conducted in the Global North show that approach‐oriented strategies associate with better psychosocial adjustment in LGBTIQ+ individuals, whereas avoidance‐oriented strategies may provide short‐term relief but are linked to poorer long‐term psychological well‐being (Lehavot 2012; Sandfort et al. 2009; Seelman et al. 2017). For instance, Kaysen et al. (2014) indicated that young adult sexual minority women experiencing internalized homophobia are more likely to use avoidance‐oriented coping strategies like self‐concealment, which can exacerbate mental health issues. In contrast, studies with younger LGB adults have shown that seeking social support or engaging in activism can foster a sense of agency and promote better psychosocial adjustment and school attainment (Toomey et al. 2018). In addition, self‐care and management of mental health have been identified as effective personal coping mechanisms. Self‐care approaches (Wolpert et al. 2019) and taking responsibility for personal well‐being and behavior (Town et al. 2022) have been shown to improve mental health outcomes in LGBTIQ+ youth – strategies that can be extended to adults or communities in Turkey.

### 1.3 The Current Study

While LGBTIQ+ people in Turkey might be frequently exposed to adverse experiences and minority stress, studies examining the associations between these experiences and the psychological consequences are very limited. However, living in a place that does not embrace protective policies against discrimination based on SOGI might adversely affect the mental health of LGBTIQ+ people (Wadsworth and Hayes‐Skelton 2015). With its historical and social structure, Bursa is a city in Turkey that contains a wide range of risk factors making life difficult for the LGBTIQ+ community. In terms of hate crimes against LGBTIQ+ people, Bursa occupied the 5th place in 2016, 8th in 2017, and 5th again in 2018 in Turkey (Kaos 2016; 2017a; 2018a). According to media monitoring reports from 2017 to 2019, Bursa's local press targeted LGBTIQ+ individuals and organizations most frequently in comparison to other cities (Kaos 2017b; 2018a; 2019c). Therefore, it is imperative to understand the lived experiences and mental health of LGBTIQ+ people who live or have lived in Bursa, as well as the association between those. It is also vital to investigate the factors protective of individuals' mental health and the assets and resources related to their resilience.

The current study was conducted with the aims of 1) investigating the discrimination experiences that LGBTIQ+ individuals face due to their SOGI, 2) examining the associations between the discrimination experiences, mental health, and resilience, and 3) amplifying the personal narratives of LGBTIQ+ community in Bursa in relation to their mental health. A mixed‐methods design was chosen to provide both breadth and depth in understanding the mental health experiences of LGBTIQ+ individuals. While quantitative methods allowed for the identification of patterns and statistical relationships between discrimination, resilience, and mental health outcomes, qualitative methods captured the lived experiences and contextual nuances that numbers alone cannot fully convey. Given the complexity of discrimination experiences and resilience processes, incorporating qualitative methods in this study was essential for capturing the nuanced, lived realities of LGBTIQ+ individuals. By integrating both approaches, this study aimed to offer a more comprehensive and nuanced understanding of the interplay between discrimination, resilience, and well‐being in this marginalized community.

We expected that perceived discrimination would be linked with poorer mental health. Mental health is a broad, multidimensional concept that encompasses not only the presence or absence of psychological symptoms but also positive aspects of functioning and well‐being (Keyes 2005). Mental well‐being refers to a state in which individuals experience positive feelings and functioning in life, including aspects such as optimism, positive relationships, autonomy, and a sense of purpose. Therefore, perceived discrimination would be associated with lower mental well‐being and higher psychological symptoms, specifically anxiety, depression, and negative self‐concept. In addition, we expected that resilience factors (i.e., personal and relational resilience, coping strategies, and self‐care) would mitigate the negative psychological impact of discrimination by buffering against psychological symptoms and promoting well‐being. We also anticipated that identity‐based lived experiences and practices (i.e., coming out, access to healthcare, self‐care practices) within this community would provide in‐depth insights into their well‐being and resilience.

## 2 Method

### 2.1 Research Design

This research was designed and implemented by the members and volunteers of the Özgür Renkler Derneği [Free Colors Association], who work in fields of the academy and civil society, by adopting a co‐production approach (Banks et al. 2018). Co‐produced research generates knowledge “with the community” instead of “on the community” and facilitates a synthesis of academic research experience and practical knowledge and “lived expertise” of the community. It aims for an egalitarian, democratic, and respect‐based interaction, as well as a positive change resulting from this process, including community empowerment and capacity building.

As co‐researchers, we decided that a convergent mixed‐method design would be suitable for addressing our research aims. In this single‐phase approach, researchers obtain both quantitative and qualitative data, analyze them separately, and then compare the results to integrate the information into a coherent whole (Creswell and Creswell 2018). We also reviewed the literature to identify established measurement tools (i.e., quantitative tools) to assess the constructs we aimed to explore, as well as formulated the open‐ended questions (i.e., qualitative tools) to explore related concepts more in‐depth through personal narratives.

### 2.2 Participants

The sample included 69 participants who identified as LGBTIQ+, aged 18–47 (*M* = 26.70, SD = 7.69). Among them, 74% of the participants reported that they live in Bursa, and 26% stated that they had lived in Bursa in the past. In regard to gender identities and intersex status, 27.9% of the participants described themselves as cis woman, 25% as cis man, 14.7% as non‐binary, 10.3% as trans man, 7.4% as trans woman, 4.4% gender‐fluid, 1.5% as agender, and 1.5% as intersex. Meanwhile, four participants (5.9%) chose the option of “other description” (e.g., queer), and one participant (1.5%) preferred not to answer. In terms of sexual orientation, 34.9% of the participants described themselves as gay, 24.6% as bisexual, 15.9% as lesbian, 11.6% as pansexual, 4.3% as demisexual, and 1.4% as asexual, whereas five participants chose the option of “other description” for their sexual orientations (e.g., heterosexual, polysexual, bicurious).

Participants had high education levels: 23.5% of the participants stated that they were undergraduate students, 41.2% held a bachelor's degree, 14.7% had a master's degree, and 5.9% held a PhD; whereas only 5.9% were high school graduates and 8.8% had an associate degree. Almost half of the participants stated that they had a full‐time (39.1%) or a part‐time (8.7%) job, whereas the rest were unemployed (23.2%) or in full‐time education (29%). The average monthly income was 5,364 TL (~765 USD; SD = 2.500,71 TL), while the incomes of 59.6% of participants were lower than the minimum wage (2800 TL).

After completing the quantitative scales, 15 participants exited the survey without providing qualitative insights into their lived experiences and practices. However, their profiles were similar to the rest of the sample, making it unlikely that their absence introduced significant bias into the findings.

### 2.3 Data Collection Tools

Data were collected through questionnaires (quantitative data) and open‐ended questions (qualitative data). All participants provided information about their demographic features, mental well‐being, psychological symptoms, resilience, and experiences of discrimination, respectively (for descriptive statistics, see Table 1).

**Table 1:** Descriptive statistics and zero‐order correlation coefficients for study variables.

Variable	1	2	3	4	5	6	7	8
1. Mental well‐being	—							
2. Anxiety	−0.53***	—						
3. Depression	−0.64***	0.80***	—					
4. Negative self‐concept	−0.56***	0.82***	0.78***	—				
5. Personal resilience	0.64***	−0.33**	−0.30*	−0.41**	—			
6. Relational resilience	0.57***	−0.35**	−0.29*	−0.34**	0.63***	—		
7. Individual discrimination	−0.39**	0.38**	0.37**	0.36**	−0.42***	−0.44***	—	
8. Group discrimination	−0.34**	0.24*	0.31*	0.25*	−0.34**	−0.42***	0.47***	—
*M*	2.09	1.95	2.39	2.09	2.4	2.36	1.68	3.16
SD	0.78	0.82	0.85	0.79	0.67	0.92	0.98	0.73
Min	0	0	0	0	1	1	0	1
Max	3.57	4	4	4	4	4	4	4

* *p* < 0.05

** *p* < 0.01

*** *p* < 0.001.

#### 2.3.1 Demographic Form

A form was used to assess participants' demographic background, such as age, SOGI, level of education, and occupation. SOGI concepts were briefly defined right below the options in the form.

#### 2.3.2 Mental Well‐Being

Participants' mental well‐being was assessed using the Warwick‐Edinburgh Mental Well‐being Scale (Tennant et al. 2007; adapted to Turkish by Keldal 2015). This scale, consisting of 12 items (e.g., “I've been feeling optimistic about the future”), focuses on the positive mental health of individuals by encompassing mental well‐being and subjective well‐being. The items were rated on a 5‐point Likert scale ranging from 0 = *Does not describe me at all* to 4 = *Describes me very well*. The total score was calculated by averaging the scores from individual items (Cronbach's *α* = 0.91).

#### 2.3.3 Psychological Symptoms

Participants' psychological symptoms were assessed through the Brief Symptom Inventory (BSI), a short form of the SCL‐90‐R (Derogatis 1992: adapted to Turkish by Sahin and Durak 1994). It includes 53 items and measures the psychological symptoms in five dimensions: anxiety, depression, negative self‐concept, somatization, and hostility. The current study focused on the symptoms related to anxiety (*n* = 13 items; e.g., “Suddenly scared for no reason”), depression (*n* = 12 items; e.g., “Feeling lonely”), and negative self‐concept (*n* = 12 items; e.g., “Feeling inferior to others”); therefore, the participants were administered only the relevant subscales. The items were scored on a 5‐point Likert scale ranging from 0 = *None* to 4 = *Advanced*. A total score for anxiety (Cronbach's *α* = 0.87), depression (Cronbach's *α* = 0.89) and negative self‐concept (12 items; Cronbach's *α* = 0.85) subscales were calculated by averaging the scores obtained from corresponding items.

#### 2.3.4 Resilience

Resilience was assessed using the Adult Resilience Measure developed by Ungar et al. (Resilience Research Centre 2018; adapted to Turkish by Arslan 2015) via 17 items in two subdimensions: personal resilience (*n* = 10 items; e.g., “I cooperate with people around me.”) and relational resilience (*n* = 7 items; e.g., “My family have usually supported me through life.”). The items were scored on a 5‐point Likert‐type scale ranging from 0 = *Does not describe me at all* to 4 = *Describes me very well*. Participants' personal resilience (Cronbach's *α* = 0.83) and relational resilience (Cronbach's *α* = 0.84) scores were calculated by averaging the corresponding items' scores.

#### 2.3.5 Perceived Discrimination

**Figure 1:**
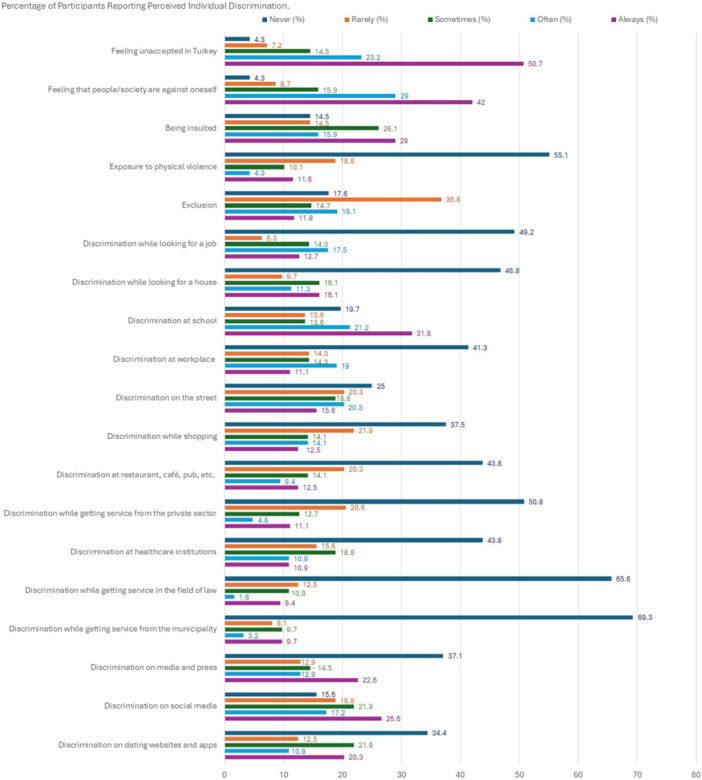
Percentage of participants reporting perceived individual discrimination.

**Figure 2:**
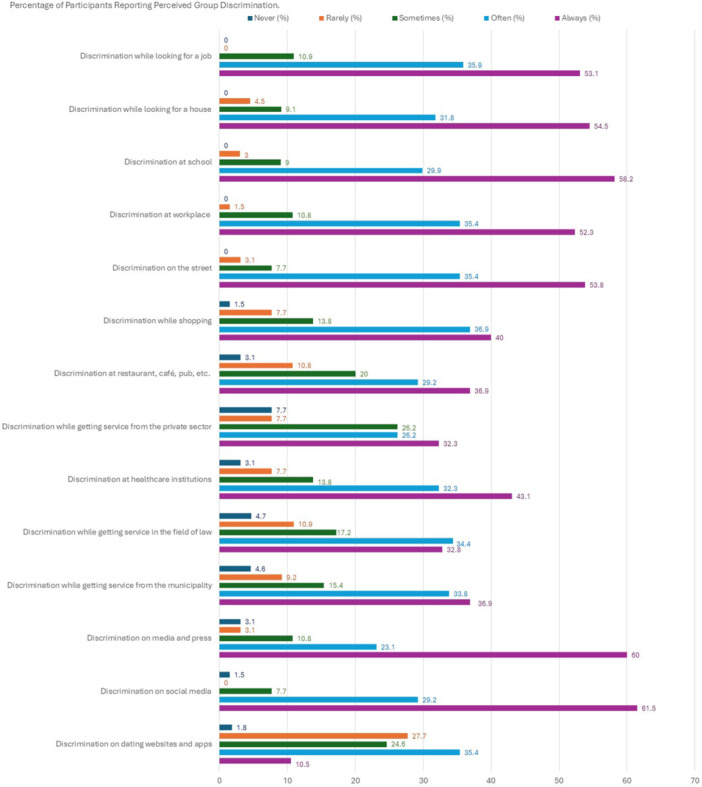
Percentage of participants reporting perceived group discrimination.

Participants' perceptions of discrimination were assessed at individual and group levels using the Perceived Individual Discrimination Scale (PIDS) and Perceived Group Discrimination Scale (PGDS) developed by Ruggiero and Taylor (1995). PIDS includes four items and assesses individual experiences of discrimination, while PGDS explores discrimination encountered by the community members via seven items. PIDS and PGDS items can be seen in Figures 1 and 2, respectively. Both scales were adapted into Turkish to measure perceived ethnic discrimination in various studies (e.g., Akbaş 2010), whereas Basar and Oz (2016) adapted these scales to measure the experiences of trans people. In this study, the abovementioned scales were adapted to LGBTIQ+ people/community. For this, we added an extra item to PIDS assessing the experience of physical assault, and eight items to PGDS assessing discrimination experiences in the following areas: places such as restaurants; during services obtained from law, municipalities, the private sector, etc.; media, social media, dating websites and apps. Also, the participants were asked to respond to PGDS items not just for the experiences of the LGBTIQ+ individuals living in Turkey but also for their own. The items were scored on a 5‐point Likert scale ranging from 0 = *Never* to 4 = *Always*; and the perceived individual (Cronbach's *α* = 0.94) and group (Cronbach's *α* = 0.94) discrimination scores were calculated by taking the average of scores obtained from corresponding items.

#### 2.3.6 Lived Experiences and Practices

The lived experiences and practices were assessed using a form developed by the LGBTIQ+ activists in Bursa. The form includes questions about coming‐out experiences, access to healthcare, and self‐care practices. In regard to their coming‐out experiences, participants were asked whether they got prepared for/before coming out and how, to whom they came out, what they encountered during/after coming out, and how they managed the process in case of a negative encounter. The participants who stated that they did not come out (yet) received other follow‐up questions such as whom they plan to come out, whether they get prepared or not, and how they feel about this process. Those who noted that they did not come out and do not plan to, were asked to share the reasons for it. Regarding healthcare, participants were asked to share their experiences when they accessed healthcare services, whether they shared their SOGI, and whether they were satisfied with the service they received, as well as their experiences related to the transition process, gynecology, or HIV test centers. Participants were also asked to share about their self‐care practices during difficult days or periods, such as the routines or actions they perform to feel better, or factors/elements that empower them.

### 2.4 Procedure

This research was approved by the Koç University Social Sciences Research.

Ethical Committee (Protocol No: 2020.387.IRB3.141). The participants were reached through convenience and snowball sampling methods. The data were collected between October and December 2020 from LGBTIQ+ adults who live/had lived in Bursa through an online survey prepared on Qualtrics. Özgür Renkler Derneği [Free Colors Association], which is based in Bursa, and the partner LGBTIQ+ institutions shared the link of the survey periodically (once in 2–3 weeks) on websites and social media accounts.

The study was completed in a single session. After filling in the consent form, participants answered the questions in the survey. Completion time was 20–30 min. After the survey was completed, the participants were thanked for their participation and informed about the contact details of the association, if they want to receive consultancy or get in contact with them.

### 2.5 Analysis Plan

To answer the research questions, the three steps of convergent design (Creswell and Creswell 2018) were followed. Accordingly, we conducted the quantitative and qualitative analyses separately, ensuring methodological rigor in each strand. The findings were then compared and integrated through a process of triangulation, identifying areas of convergence, divergence, and complementarity. Specifically, we used a side‐by‐side comparison strategy, where key quantitative results were juxtaposed with qualitative content categories to explore how they reinforced or nuanced each other. Points of alignment were used to strengthen overall interpretations, while discrepancies were examined to provide deeper insights into the complexity of the lived experiences of the participants. This integration was carried out in the discussion section, where we synthesized the findings to develop a more comprehensive understanding, drawing on both numerical trends and participants' lived experiences.

The quantitative analyses were performed using IBM SPSS v20. The relations between variables were calculated with the Pearson correlation coefficient. The role of perceived discrimination and resilience in mental well‐being and psychological symptoms was examined via multivariate regressions. For each dependent variable (mental well‐being, anxiety, depression, and negative self‐concept), demographic variables (age, level of education [1=high school graduate, 6= Ph.D.]) were entered into the model as control variables, then perceived individual and group discrimination were added in the second step, and personal and relational resilience were added in the third step.

The qualitative data were evaluated using qualitative content analysis (QCA) (Jaspal 2020; Vaismoradi and Snelgrove 2019) via NVivo 12. In QCA, “the analyst organizes a large corpus of data into [codes and] categories, which reflect the content of the data or text” (Jaspal 2020, 289). In this study, content categories were developed to systematically capture the manifest, or surface‐level, dimensions of participants' accounts. These categories provide a transparent and structured representation of the descriptive findings, in line with the study's objectives (Vaismoradi and Snelgrove 2019). While superordinate categories function similarly to overarching themes and content categories resemble sub‐themes, our analytic approach differs from thematic analysis by focusing on the content, frequency, and diversity of codes rather than on latent meaning‐making processes. The analysis procedure started with becoming familiar with the data, as the first author read the dataset several times. As a result of this process, the initial codes were generated. Then, the first author and the other authors ran back over these initial codes and produced the secondary codes. In order to demonstrate the diversity in the codes, the frequencies were also determined. Similar patterns among the secondary codes were identified, and the content categories were produced. In the final stage, superordinate categories were created based on the interconnections among individual content categories. During the analysis, both inductive and deductive approaches were implemented. After the content categories and superordinate categories were finalized, the sample quotes were chosen, and the content was reviewed for the last time. While presenting the results through the quotes by the participants, their ID numbers starting with “P,” ages, gender identities, and sexual orientations have been provided. Although rarely, the quotes were edited out to correct spelling and grammar. The information added by the authors to clarify the meaning is presented between square brackets.

### 2.6 Positionality Statement

All authors are from Turkey and are highly familiar with the local and global sociopolitical context and dynamics in relation to sexual and gender minorities. BK is an academic co‐researcher who has been involved in LGBTIQ+ activism. She is passionate about and committed to researching mental health and well‐being in marginalized communities and translating this knowledge into ways to improve their quality of life. DG is a community co‐researcher, a trans woman and HIV+ activist, working as a human rights monitoring expert. SÖ is a community co‐researcher, a human and animal rights defender, and a non‐binary illustrator who uses their art to raise awareness on LGBTIQ+ rights. DEŞ and UŞ are academic co‐researchers whose work focuses on gender, sexuality, queer theory, and critical psychology.

## 3 Results

### 3.1 Quantitative Data

On average, participants reported moderate levels of mental well‐being, psychological symptoms, resilience, and perceived individual discrimination, as well as a high level of perceived group discrimination for LGBTIQ+ individuals living in Turkey (see Table 1).

#### 3.1.1 Perceived Discrimination

Figure 1 presents the percentage of participants reporting perceived individual discrimination experiences (a text‐based version of these data is provided in Supporting Information S1 for accessibility). The findings showed that participants often encounter discrimination in every aspect of their lives due to their LGBTIQ+ identity. The majority (73.9%) noted that they often or always feel unaccepted in Turkey. Similarly, 71% stated that they often or always feel that people and/or society are against them. Being mocked and/or insulted was another form of discrimination that is frequently experienced, where 85.5% of the participants stated that they were exposed to this type of experience at least once, while almost half (44.9%) reported that they often or always encounter similar incidents. With regard to being exposed to physical violence, whereas 44.9% of the participants selected the option “at least once,” 15.9% reported that they are often or always exposed to physical violence. While a clear majority (82.4%) noted that they were left out and excluded at least once, 30.9% reported that they often or always go through such experiences. Approximately half of the participants stated that they have encountered discrimination while looking for a house (53.2%) or a job (50.8%). Concerning the same point, 30% noted that they often or always face discrimination in these areas. Four out of every five participants (80.4%) stated that they were subjected to discrimination at school. Half of them (53%) expressed that such experiences had often or always recurred. More than half of the participants (58.7%) were exposed to discrimination at the workplace at least once. The rate of participants who are frequently or always discriminated against at work was 30.1%. Three out of every four (75%) participants reported that they faced discrimination on the street at least once, and 35.9% noted that they often or always experience that. In addition, 62.5% of participants expressed that they experienced discrimination while shopping at least once. One out of every four participants (26.6%), on the other hand, mentioned that they often or always experience discrimination while shopping. At least half of the participants (56.2%) stated that they were discriminated against at restaurants, cafés, pubs, or other similar places at least once, and 21.9% noted that they often or always experience this form of discrimination. Similarly, half of the participants (49.2%) stated that they have faced discrimination while getting service from the private sector (e.g., gym, bank, etc.). The rate of participants who are often or always discriminated against in these areas was 15.9%. Whereas the rate of participants who have encountered discrimination at healthcare institutions was 56.2%, 21.8% stated that they often or always experience this form of discrimination. One in every 3 participants stated that they were exposed to discrimination at least once while getting service in the field of law (34.4%) or from the municipality (30.4%), and one out of every 10 participants noted that the discrimination in these areas often or always took place. The rate of participants who have encountered discrimination on press and media was 62.9%. However, the ones who are often or always discriminated against in this field constituted 35.5%. Social media was reported as another area in which discrimination has frequently taken place: 84.4% of participants reported that they experienced discrimination on social media at least once, and 43.8% mentioned that it often or always happens. Three out of every five participants (65.6%) mentioned that they have experienced discrimination on dating websites and applications, and 31.2% noted that it often or always takes place.

Figure 2 shows the percentage of participants reporting discrimination against LGBTIQ+ group (for a text‐based version of the data, see Supporting Information S1). The participants reported that LGBTIQ+ people living in Turkey are frequently exposed to discrimination. According to the participants, varying between the rates of 75% and 90%, LGBTIQ+ people are often or always exposed to discrimination while looking for a house or a job, at school or workplace, on the street, while shopping, at healthcare institutions, in the media and press, or on social media. Moreover, the participants thought that there were no LGBTIQ+ people who had not encountered discrimination while looking for a house or a job, at school, at the workplace, or on the street. The perceived discrimination regarding other areas, reported by the participants, was also quite high. Accordingly, participants, varying between the rates of 59% and 75%, reported that LGBTIQ+ individuals often and always encounter discrimination at places such as restaurants, cafés, pubs, etc., while getting service from the private sector (e.g., gym, bank), while getting service from the municipality or in the field of law, and on dating websites and apps.

Ten participants shared their experiences of discrimination, which were not included in the questionnaire, by choosing the option of “other.” These experiences included sexual assault, being in constant fear of life, feeling insecure on the streets at night, being excluded by the family, being kicked out from school groups by schoolmates, being swindled, being slandered, exerting greater efforts to fit in socially than their (heteronormative, cis) peers, and feeling as though others' eyes were drilling into them. One participant who identifies as a trans woman stated that they had been discriminated against due to their penis and not been acknowledged as a lesbian. Another participant shared that they were tortured by the law enforcement officers and sued because of the officer who broke their finger during the torture. Another participant stated that they were exposed to meaningless questions interrogating their sexual orientation and treated like a “circus animal” by the heterosexual people who know about their sexual orientation. One participant, in addition, noted that they have not been discriminated against because they do not share their sexual orientation with anyone, but also that they are sure of being discriminated against if they come out.

#### 3.1.2 The Associations Between Perceived Discrimination and Mental Health

As presented in Table 1, perceived individual and group discriminations positively correlated with psychological symptoms and negatively correlated with mental well‐being and resilience. Meanwhile, psychological symptoms were negatively correlated with both mental well‐being and resilience. Mental well‐being and resilience, on the other hand, positively correlated with each other.

#### 3.1.3 The Role of Resilience

The role of perceived individual and group discriminations on mental well‐being, anxiety, depression, and negative self‐concept, as well as the role of personal and relational resilience in this process, were examined using hierarchical regression models (for statistics, see Table 2). In the first step of the analysis, demographic variables (age, level of education) were included as control variables. Perceived individual and group discrimination in the second step and personal and relational resilience in the third step were added to the model. Each dependent variable (mental well‐being, anxiety, depression, and negative self‐concept) was analyzed separately.

**Table 2:** Hierarchical regression analysis of the variables predicting mental well‐being and psychological symptoms (*n* = 69).

Variables	Mental well‐being			Anxiety			Depression			Negative self‐concept		
	*β*	*R* ^2^	Δ*R* ^2^	*β*	*R* ^2^	Δ*R* ^2^	*β*	*R* ^2^	Δ*R* ^2^	*β*	*R* ^2^	Δ*R* ^2^
1st step		0.00	0.00					0.03	0.03		0.02	0.02
Age	−0.02			−0.08			−0.13			−0.13		
Education	0.06			0.12	0.02	0.02	−0.06			−0.01		
2nd step		0.18	0.18**					0.18	0.16**		0.17	0.15*
Age	−0.01			−0.13			−0.16			−0.18		
Education	0.03			0.16			−0.03			0.03		
Perceived individual discrimination	−0.29*			0.39**			0.32*			0.36**		
Perceived group discrimination	−0.20			0.04	0.18	0.16**	0.14			0.05		
3rd step		0.47	0.28***					0.21	0.02		0.25	0.08*
Age	0.01			−0.13			−0.16			−0.19		
Education	−0.03			0.18			−0.02			0.05		
Perceived individual discrimination	−0.08			0.30*			0.26			0.26		
Perceived group discrimination	−0.05			−0.03			0.10			−0.02		
Personal resilience	0.45**			−0.13			−0.14			−0.28		
Relational resilience	0.23			−0.16	0.23	0.05	−0.05			−0.06		

* *p* < 0.05

** *p* < 0.01

*** *p* < 0.001.

##### 3.1.3.1 Mental Well‐Being

In Step 1, when the demographic variables were added, the model did not explain mental well‐being, *F*(2,64) = 0.10, *p* = 0.90. In Step 2, after adding the experiences of discrimination, the model improved significantly and explained 18% of the total variance, *F*(2,62) = 6.89, *p* = 0.002. Perceived individual discrimination significantly predicted lower levels of mental well‐being. In Step 3, the addition of resilience variables significantly contributed to the model, which explained 47% of the total variance, *F*(2,60) = 15.90, *p* < 0.001. Personal resilience predicted higher levels of mental well‐being. The negative association between perceived individual discrimination and mental well‐being disappeared.

##### 3.1.3.2 Anxiety

In Step 1, the model including demographic variables could not explain anxiety to a significant extent, *F*(2,64) = 0.51, *p* = 0.60. After the experiences of discrimination were added, the model significantly improved and explained 18% of the total variance, *F*(2,62) = 6.19, *p* = 0.004. Perceived individual discrimination significantly predicted higher levels of anxiety. In Step 3, the inclusion of resilience variables did not significantly contribute to the model, *F*(2,60) = 1.96, *p* = 0.15. However, the beta value of perceived individual discrimination decreased from 0.39 to 0.30.

##### 3.1.3.3 Depression

The demographic variables were added to the model in Step 1, but the model could not explain depression to a significant extent, *F*(2,64) = 0.82, *p* = 0.45. When the experiences of discrimination were included, the model showed significant improvement and explained 18% of the total variance, *F*(2,62) = 6.069, *p* = 0.004. Perceived individual discrimination significantly predicted higher levels of depression. Adding resilience variables did not significantly contribute to the model, *F*(2,60) = 0.79, *p* = .49, however, the beta value for perceived individual discrimination decreased from 0.32 to 0.26.

##### 3.1.3.4 Negative Self‐Concept

After entering demographic variables into the model, it could not explain negative self‐concept, *F*(2,64) = 0.59, *p* = 0.56. When the experiences of discrimination were added in Step 2, however, the model improved significantly and explained 17% of the total variance, *F*(2,62) = 5.52, *p* = 0.01. Perceived individual discrimination significantly predicted higher levels of negative self‐concept. Entering resilience variables in the third step significantly contributed to the model, which explained 25% of the total variance, *F*(2,60) = 3.17, *p* = 0.049. Personal resilience marginally predicted lower levels of negative self‐concept. Also, the beta value of perceived individual discrimination decreased from 0.36 to 0.26 and became insignificant.

### 3.2 Qualitative Data

The qualitative responses regarding the lived experiences of participants were analyzed across three superordinate categories: Coming out, access to healthcare, and self‐care practices. Table 3 shows the codes and content categories that constitute each superordinate category, as well as the frequency of codes.

**Table 3:** Categories, codes, and frequencies observed.

Superordinate category	Content category	Code	Frequency^a^
Coming Out	Positive experiences	Being supported	24
		Being accepted	6
		Feeling empowered, self‐esteemed, or relaxed	3
		Building stronger relationships	4
	Negative experiences	Phobia, verbal abuse, exclusion, peer bullying	20
		Friend's cutting off the communication	8
		Obligation of concealing identity	3
		Identity revealed unconsented	3
	Coping with coming out process	Becoming conscious and increasing awareness	6
		Keeping in touch with people who accept them	3
		Defending themselves	3
		Ignoring phobic attitudes	2
		Professional psychological support	2
		Never mentioning again	2
		Letting things slide	1
		Solidarity with other LGBTIQ+ people at school	1
Access to healthcare	Competence of services	Insufficient/non‐inclusive for LGBTIQ+ people	33
		Sufficient for LGBTIQ+ people	2
		Unequal access among LGBTIQ+ people	1
	Revealing LGBTIQ+ identity	Concealing LGBTI+ identity	28
		Revealing only when it is necessary	5
		Having a visible identity	3
		Preferring to share	1
		Revealing only to mental health professionals	1
		Revealing as a reaction to patriarchal questions	1
	Treatment encountered	Experience of prejudice, phobia, maltreatment	20
		Experience from doctor to doctor	5
		Difference between public and private hospitals	2
		Not encountered any problem	6
Self‐care practices	Social circle and support	Contacting with their social circle	15
		Spending time with pets or street animals	4
		Talking with mental health professional	2
	Coping strategies and resources	Doing activities	33
		Comforting or calming oneself down	11
		Dreaming about a better future	6
		Believing themselves	6
		Empowering resources	4
		Religion	1
	Maladaptive responses	Using alcohol	5
		Self‐isolation	5
		Sleeping to avoid issues	5

a Number of times endorsed.

#### 3.2.1 Coming Out

Forty‐four participants shared that they had come out at least once. Among them, 34 participants stated that they came out to their close friends, 20 to their parents, 17 to their siblings, and 19 to their relatives. Also, 18 participants stated that they came out at school and 12 at the workplace. In addition, three participants stated that they have not come out yet but were planning to.

Six participants shared that they did not come out and were not planning to, because they were worried about being excluded, their lives getting ruined, being disallowed by the family or peripheral cultures. The following quote illustrates this:

Because it doesn't seem very possible that my family accepts me and understands how I feel, due to the place they grew up, I am afraid of my family's reaction or being apart from my family, conflicting with them. I have a desire to have my own financial power, to form a life for myself, and live my life even if I don't fully express my identity.

(P44, 29, trans man, lesbian)

Further analysis of participants' responses regarding their coming‐out experiences revealed three content categories: positive experiences, negative experiences, and coping with coming out process.

##### 3.2.1.1 Positive Experiences

Eighteen participants shared that they encountered supportive attitudes by the people to whom they came out, particularly their friends (*n* = 12). Three participants shared that their friends responded like “we already knew,” “we guessed,” “we were aware of that.” The support of family members, especially siblings and the mother, was also mentioned (*n* = 8). In addition, four participants stated that they had positive reactions from everyone to whom they came out, including relatives and teachers. Two participants, on the other hand, stated that their mothers, even though they have been supportive, see these identities as a “phase.” The following quote exemplifies this:

Even though it has been five years since my coming‐out, my mother still thinks that it is a phase. But she is a very supportive woman except this issue. So, we don't talk about this issue, and we're good.

(P41, 18, cis woman, lesbian)

Being accepted is another aspect highlighted by the participants (*n* = 6). One participant stated that their friends have accepted them as they are and that this is how it should be. One trans participant shared that their family reacted too strongly due to the health‐related concerns and fears about the (transition) surgeries, but started to accept in the course of time. Another participant shared that they have not been accepted by the father's side, as they were “extremely traditionalist and religious,” but the mother's side, who embrace a more urban lifestyle, has accepted the situation:

Since the father's side is extremely traditionalist and religious, they couldn't accept. But the mother's side is more urban; since they have seen different lifestyles (this is my interpretation), they accepted. Even though I have some troubles with the mother's side at first, they chose me. I am happy about it.

(P14, 25, non‐binary, gay)

Seven participants mentioned the impacts of favorable coming‐out experiences, such as feeling empowered, self‐esteemed, or relaxed (*n* = 3), or building stronger relationships (*n* = 4). Two of them also highlighted that they actually feel very lucky about not having any negative experience. The following quote illustrates this:

I didn't experience anything negative, but I interpret it as a chance. It won't be an easy thing as long as there are people who died/are killed because of this reason. My mother's acceptance process is still going on a little bit hard, but I think I can overcome this too. My siblings told me that they will always back me up. This is a huge reason to feel confident.

(P63, 23, trans man, demisexual)

##### 3.2.1.2 Negative Experiences

Thirty‐four participants shared their coming out experiences that did not conclude positively in terms of negative reactions encountered.

The participants stated that they have been often exposed to experiences such as homophobia, transphobia, swearing and insults, exclusion, or peer bullying (*n* = 20). One of the participants shared that their parents threatened them not to let them go to university, and that they had to lie by saying that they would “normalize.” Another participant stated that when they came out to their mother, they had a positive reaction at first but the mother changed the attitude due to reading some misleading sources on the internet. The following quotes illustrate these situations:

My family tried to make me surrender by threatening with not letting me go to the university. I lied to go to school. Because they think of it as an abnormal situation, I got over it by promising to ‘normalize.’ Now, I am at the university and don't plan to come out one more time until I get a profession.

(P19, 19, cis woman, lesbian)

I came out to my mother. The first day I told her, she reacted better than I expected. She told me that I am her son in any case. In the following days, the condition changed a little. She searched homosexuality on the internet and read misleading sources. She started to tell me that it is a disease and if I would have said earlier, I might have been treated.

(P16, 28, cis man, gay)

Three participants, who had come out in their high school years, shared that they were exposed to peer bullying, insults, and discrimination. Another participant, who came out to one of their high school friends, said that their friend has seen them as a “wannabe.” An intersex participant expressed that they have been treated as a freak and excluded since their childhood. A bisexual cis woman participant stated that they have been sexually objectified by cis‐heterosexual men. Another participant noted that their close friend's mother, after learning their LGBTIQ+ identity, tried to stop them from seeing each other.

Another frequently mentioned reaction was the friend group's moving away or cutting off communication (*n* = 8). The reason for friends choosing to move away was mostly their homophobia, and sometimes being exposed to other people's homophobia. Two participants stated that after their coming out, this issue has never been talked about again, or people to whom they came out were not eager to listen. For example:

Earlier, I had a cis‐heterosexual woman friend to whom I was close once. She was close to the main opposition, liberal in her own way, embracing an egalitarian view. So, one day I suddenly came out to her. I felt something weird and we drifted apart in the following days. I didn't question or go after it. I saw what's going on and I moved away too.

(P65, 28, cis woman, bisexual)

Three participants shared their experiences of being obliged to hide their identities due to the discrimination they have been exposed to at school or in professional life. The following quote illustrates this:

I am a 24‐year‐old trans man, at the end of my [transition] process. In 2016, I graduated from university and began to work. Actually, my own profession is finance/accounting. But, before getting started with the transition process, I couldn't find a job in my own area because of [other people] thinking that ‘Woman or a man? No idea.’ I have been employed as a cashier, gas pump attendant, waitperson but I preferred corporate [life] in the end. And I can say that if you have to or want to work, you have to hide something or walk softly. When needed, I hid it from many people. My attitudes and behaviours, my feelings, my clothes are included! (…) I had to make concessions on several things. I wished to practice my own profession and to work in a place that openly accepts these, but I was obliged and complied. I kept being silent when they call me with my dead name, when they said, ‘Why is your hair short?’ or ‘Why are you like a man?’

(P47, 24, trans man, demisexual)

Three participants, on the other hand, stated that their coming‐outs took place without their consent. The following quotes illustrate this:

I came out with doctors' diagnosis, not by myself, when I was a child.

(P71, 39, intersex, asexual)

When my ex‐girlfriend's mother learned about us, she told my parents that.

(P50, 21, cis woman, bisexual)

##### 3.2.1.3 Coping With Coming Out Process

Twenty‐two participants shared their experiences of coping with the coming‐out process. They stated that coming out has been a psychologically overwhelming and difficult process, and one might feel alone, excluded, intolerant, and damaged. How they have reacted to negative encounters has been shaped by their personalities and the contextual conditions. For instance, six participants shared that becoming conscious and increasing awareness have helped them to manage these processes:

I managed [the process] by channelling my energy to learn more. I learned more things about LGBTIQ+ issue, and this empowered me.

(P65, 28, cis woman, bisexual)

Three participants shared that they have kept in touch with the people who accept them, or they have made friends with people who accept them:

My way of coping with the negative situation that I faced in high school was making new friends who will accept me. I'm glad it happened because these friendships have always made me happy.

(P08, 20, queer, gay)

A participant, who experienced peer bullying in high school, shared that they managed the process in solidarity with other LGBTIQ+ peers. Two participants noted getting professional psychological support, which they found helpful. While two participants expressed that they have tuned out or ignored the phobic attitudes, three participants stated that they have defended themselves and quieted the others down:

When one defends oneself, even an ultra‐phobic person backs down and leaves that attitude aside.

(P14, 25, non‐binary, gay)

Other coping methods the participants have mentioned were letting things slide (*n* = 1) and never mentioning again (*n* = 2).

#### 3.2.2 Access to Healthcare

The analysis of participants' responses regarding healthcare services revealed three content categories: *Competence of services, revealing LGBTIQ+ identity*, and *treatment encountered*.

##### 3.2.2.1 Competence of Services

Most of the participants expressed that the healthcare services are not sufficient for and inclusive of LGBTIQ+ people (*n* = 33). It is also highlighted that this insufficiency includes the transition process and anonymous HIV test centers.

While one of the participants said that healthcare services are aimed at cis and heterosexual individuals, eight participants pointed out the discrimination and negative attitudes encountered while getting healthcare services. Another participant, additionally, emphasized the necessity of training for healthcare professionals regarding their attitudes towards LGBTIQ+ people. The following quote illustrates this:

Healthcare services, in Turkey and in Bursa province, are not LGBTI‐inclusive. I even think that there is a huge prejudice. I don't think they have any study/training about it in their education. If there was a standard training about this, I think, we wouldn't be obliged to face the doctors who act very differently.

(P65, 28, cis woman, bisexual)

While seven participants stated that they do not know about this topic or cannot comment on this since they do not get healthcare services, two participants noted that they think healthcare services are sufficient for and inclusive of LGBTIQ+. One participant mentioned that the quality of healthcare services changes according to the person getting the service. This, according to the participant, might indicate that certain groups under the LGBTIQ+ umbrella get less (or more) qualified healthcare services in comparison to the others.

##### 3.2.2.2 Revealing LGBTIQ+ Identity

Most of the participants stated that they do not share their LGBTIQ+ identity while getting healthcare services (*n* = 28). Two of them expressed that they experience stress and anxiety about revealing their LGBTIQ+ identity:

Gay or not, I think a doctor should approach everyone in the same way. For this reason, I have never mentioned my sexual identity before. But there have been times that I got stressed over the idea of the necessity to mention it.

(P16, 28, cis man, gay)

Three participants stated that they have visible identities, and it gets noticed by healthcare professionals. Therefore, they have been exposed to professionals' mocking and discriminatory behaviors:

People already understand it without the necessity to share my identity. Mostly, it is the discussions of ‘Are you a girl or a boy?’ I witness that they point at me and talk about.

(P31, 28, non‐binary, lesbian)

Five participants stated that they share their LGBTIQ+ identity when it is necessary. One participant, on the other hand, expressed that they prefer to share it, thinking that the healthcare professionals need to be informed about this issue. Another participant noted that they share their identity only with their psychiatrist. One participant, who stated that they generally do not share their identity, noted that they come out as a reaction to the patriarchal questions they encounter while getting gynecology service:

No, I don't share in general. But when I come across a doctor asking, ‘Are you married?’, during the gynaecology examination, I sometimes get angry and explain it.

(P65, 28, cis woman, bisexual)

##### 3.2.2.3 Treatment Encountered

Most of the participants stated that they have encountered prejudice, phobia, and maltreatment due to their LGBTIQ+ identities while getting healthcare services, such as mocking attitudes, prejudiced questions, disrespectful gaze and speech, harsh approaches, insults, forced surgery, and even being kicked out of the examination room (*n* = 20). The following quotes illustrate this:

When my identity is known, healthcare professionals tend to keep their distance or act more insistently about learning my private information.

(P03, 25, trans woman, pansexual)

I had to share my sexual identity at a healthcare institution a couple of times. And I was treated as a disgusting garbage bag. Even when I told it to a professor at a reputable public hospital thinking that I need to share my sexual identity for medical reasons, they kicked me out of their room.

(P27, 39, cis man, gay)

Five participants stated that the way they have been treated changes from doctor to doctor, some of whom are positive and supportive, and some others have negative attitudes. One participant, additionally, noted that the state has no policies to protect them from negative attitudes:

The general approach is to ignore LGBTIQ+s in every possible way and to expect them [to take the consequences]. Because the state doesn't have a policy encompassing them [being protective of them]. The doctor or healthcare professional takes the initiative and uses their right to help.

(P27, 39, cis man, gay)

Two participants emphasized the difference between public and private hospitals and stated that they chose private hospitals so as not to encounter discrimination. Six participants said that they did not encounter any problem while getting healthcare services. One of them stated that they completed the transition process without an issue. Another participant, on the other hand, expressed that even though they haven't had any negative experience, they were still afraid to have one in the future.

#### 3.2.3 Self‐Care Practices

The analysis of participants' responses regarding the self‐care practices in difficult days or periods revealed two content categories: *social circle and support*, and *coping strategies and resources*.

##### 3.2.3.1 Social Circle and Support

Fifteen participants stated that they get in contact with their social circle and/or get support in order to feel good during difficult days or periods, such as their friends, partners, or family members:

When I share with my friends what I live through, it empowers me to feel that they stand by me in every condition.

(P08, 20, queer, gay)

Four participants, on the other hand, shared that they spend time with pets or street animals and that taking care of them makes them feel good. Two participants stated sharing their problems with their therapist or psychiatrist, while another expressed the importance of avoiding isolation and ensuring they share their problems with someone.

If I am going through something very difficult, I write or call my therapist and they get back to me immediately. This communication empowers me.

(P18, 19, cis woman, demisexual)

##### 3.2.3.2 Coping Strategies and Resources

Participants mentioned various approach‐oriented and avoidance‐oriented ways and resources to cope with difficult days and periods. Among these, the ways of comforting or calming oneself down were commonly shared (*n* = 11), such as crying to comfort oneself and feel better (*n* = 6), taking a warm shower (*n* = 3), and moving away from the issue (*n* = 2). The following quotes illustrate this:

It feels good to move away from the problem a little bit. […] I experienced that I cool down to face the problem later, and I may get more prone to make rational decisions by seeing it more clearly.

(P65, 28, cis woman, bisexual)

Hugging my cats and crying give me relief.

(P51, 34, gender fluid, pansexual)

Most of the participants reported engaging in various activities to feel better during difficult days or periods (*n* = 33). Among these activities, the most frequently mentioned ones were listening to music (*n* = 11), reading a book (*n* = 5), watching TV series and/or movies (*n* = 5), and walking outdoors (*n* = 5). Other less frequently mentioned activities were playing games, writing, yoga or meditation, trying new recipes, doing chores, playing guitar, and having sex.

Six participants stated that dreaming about the future and believing in better days to come empowers them. Six participants, on the other hand, stated that what empowers them is themselves, their own motivations, and their determination to pick themselves up. For example:

I think about the experiences that I will have in the future, about what I want to do. I think that I have many more things to see.

(P62, 21, gender‐fluid, pansexual)

One participant stated that they find strength in their religion, while four participants stated that they benefit from empowering resources. Among these resources, there were the talks by feminist activists, motivating stories or movies, the lives of other trans people, and previously‐read and marked books:

Listening to and watching other trans people on the internet. Listening to their achievements, life stories, pains always empowers me. I am very happy that we are such beautiful people.

(P53, 18, trans woman, bisexual)

##### 3.2.3.3 Maladaptive Responses

Some participants described a range of strategies, which may reflect maladaptive ways of managing to stress, such as using alcohol (*n* = 5), sleeping (*n* = 5), and self‐isolation (*n* = 5). The following quotes illustrate this:

I sleep because I can't deal with the social pressure and difficulties of being a trans [gender person]. I can only escape by sleeping.

(P26, 18, trans woman, pansexual)

I prefer to be alone. This could last a week or even three years.

(P25, 24, cis woman, lesbian)

I get drunk.

(P7, 30, non‐binary, bisexual)

## 4 Discussion

### 4.1 Discrimination

This study highlights that minority stress is a profound and pervasive experience among participants. Discrimination occurs in nearly every aspect of their lives, systematically restricting access to fundamental human rights such as education, employment, and healthcare. These practices of exclusion and discrimination have prevented LGBTIQ+ individuals from fully participating in life in Bursa.

Both quantitative and qualitative findings underscore the significant educational discrimination participants faced throughout their schooling. Peer bullying and systemic discrimination in high school created particularly challenging experiences in our sample, especially for those who visibly and/or openly expressed their SOGI. These findings align with previous research, which highlights that LGBTIQ+ individuals experience substantial direct and indirect discrimination in education, exacerbated by societal stigmatization and a lack of inclusive policies (e.g., Cakmak 2022; Lambdaistanbul 2010; Paceley et al. 2017). Such systemic barriers often discourage individuals from reporting grievances, as they perceive the justice system as ineffective in addressing their concerns (Yilmaz and Gocmen 2015). Addressing these challenges requires implementing policies that reduce peer bullying and prevent discriminatory behavior among school staff. However, a narrative review of the literature (Coskun 2024) indicates that LGBTIQ+ studies in educational research in Turkey are extremely scarce, with little to no guidance on necessary improvements in curriculum, teacher training, pedagogical approaches, or educational policies concerning LGBTQ+ students and staff. Therefore, there is an urgent need for measures to uphold human rights and inclusive practice for sexual and gender minorities in educational settings in Turkey. Research conducted in Global North suggests that supportive interventions and whole‐school approaches (Day et al. 2016; McDermott et al. 2023), inclusive policies and enumerated protections (Russell et al. 2021), professional development for school personnel (McDermott et al. 2023; Russell et al. 2021), and curriculum inclusivity (Harris et al. 2021) promote a positive school climate where everyone can thrive and reduce homophobic and/or transphobic bullying and discrimination.

Discrimination also extends into participants' professional lives, with more than half reporting workplace discrimination at least once, amounting to a violation of their right to work. These findings are consistent with research highlighting the pervasive nature of workplace discrimination faced by LGBTIQ+ individuals in Turkey and globally. Studies in Turkey have shown that LGBTIQ+ employees frequently experience both direct and indirect discrimination, including being marginalized, excluded, or compelled to hide their identities to avoid mistreatment (e.g., Ozdemir and Acikgoz 2024). Although Turkish Labor Law prohibits discrimination on grounds such as gender or “similar” reasons, it does not explicitly include sexual orientation or gender identity (Ozdemir and Acikgoz 2024), leaving LGBTIQ+ employees vulnerable to systemic exclusion. This lack of explicit protections forces many individuals to adopt coping mechanisms such as concealing their identities, which can lead to heightened stress and reduced job satisfaction. Similar patterns have been observed internationally; for instance, research indicates that anticipated discrimination often leads LGBTIQ+ employees to suppress their identities (e.g., Stonewall 2025), exacerbating feelings of isolation and minority stress (Frost and Meyer 2023). This underscores the urgent need for legal reforms to establish protections against discrimination. In addition to legislative action, international studies indicate that compulsory anti‐bias training for employees and managers is necessary to foster inclusive workplaces and reduce bullying and discrimination (Carter et al. 2020).

Healthcare discrimination emerged as another significant issue. Many participants reported facing prejudice, phobia, and mistreatment due to their SOGI when seeking medical services, leading them to either conceal their identity or experience significant stress. These findings are consistent with research highlighting systemic barriers in Turkey's healthcare system, where LGBTIQ+ individuals often encounter discriminatory attitudes from healthcare providers, resulting in avoidance of care and unmet health needs (Uysal Toraman and Agartioglu Kundakci 2020; Ercan Sahin et al. 2020). This aligns with broader international evidence showing that healthcare discrimination leads to poorer physical and mental health outcomes among LGBTIQ+ populations (Matsutaka et al. 2024). Furthermore, a majority of participants found healthcare services inadequate and exclusionary toward LGBTIQ+ individuals. Denial of access to healthcare constitutes a serious human rights violation, as recognized by global frameworks such as the Yogyakarta Principles. In Turkey, the lack of inclusive policies and training for healthcare professionals perpetuates these disparities. Participants' experiences further highlight systemic challenges, including difficulties encountered during gender transition processes and the inadequate promotion of anonymous HIV testing centers. These issues are compounded by persistent HIV‐related stigma, which discourages individuals from seeking timely testing and treatment. Research suggests that culturally sensitive training programs for medical staff can significantly reduce biases and improve the quality of care for marginalized groups. For example, the PRIDE program has been shown to enhance healthcare providers' competency in addressing the needs of LGBTIQ+ patients by fostering empathy and reducing prejudice (Matsutaka et al. 2024).

Notably, participants expressed that no LGBTIQ+ individual in Turkey remains untouched by discrimination—whether at home, work, school, or in public spaces. In this context of widespread discrimination, some individuals are unable to disclose their identities, further hindering their full participation in society. One participant articulated this concern by stating, “I do not want to ruin my life by coming out.” This sentiment reflects broader findings that concealment of identity is a common survival mechanism among marginalized groups facing systemic oppression (ERA LGBTI 2014; Frost and Meyer 2023).

### 4.2 Mental Health

Consistent with previous research (Basar and Oz 2016; Douglass and Conlin 2020; Plöderl and Tremblay 2015), this study found that discrimination significantly predicted poorer mental health outcomes in LGBTIQ+ individuals, including lower mental well‐being and heightened psychological symptoms. Notably, perceived individual discrimination emerged as a stronger predictor than perceived group discrimination. While group discrimination was initially associated with mental well‐being and psychological symptoms, its significance diminished when individual experiences were considered, suggesting that direct personal encounters with discrimination exert a more immediate psychological toll.

Findings revealed that personal resilience played a crucial role in mitigating the negative effects of individual discrimination on mental well‐being and negative self‐concept. When personal resilience was accounted for, perceived individual discrimination lost its significance in predicting mental well‐being and negative self‐concept, and its predictive strength for anxiety and depression also weakened. While relational resilience was independently associated with mental health, its influence disappeared when personal resilience was considered. This contradicts existing literature emphasizing the protective role of social support in LGBTIQ+ individuals' coping strategies (Atalay 2019; Basar and Oz 2016), as well as the qualitative findings of this study. A possible explanation is that the relational resilience measure captured not only interpersonal support but also broader societal and cultural attitudes. Given that many participants felt socially marginalized and unaccepted, these broader contextual factors may have overshadowed the benefits of close social networks.

Lived experiences and self‐care practices reported by the participants further revealed a complex interplay between personal resilience and relational resilience, and coping strategies. The qualitative findings highlight both approach‐oriented and avoidance‐oriented coping strategies, each with distinct implications for the long‐term well‐being of LGBTIQ+ individuals. Personal resilience emerged as a significant factor in how participants navigated the challenges of coming out and coping with discrimination. For example, participants who reported becoming more conscious and increasing awareness of their experiences demonstrated an approach‐oriented coping strategy. This aligns with research suggesting that self‐awareness and engaging in activism are crucial components of resilience in LGBTIQ+ populations, helping individuals to manage minority stressors effectively (e.g., Toomey et al. 2018). Similarly, participants who found strength in their own motivations and determination reflected that self‐agency plays a central role in overcoming adversity (e.g., Dufner et al. 2019).

Relational resilience also played a pivotal role, as many participants relied on their social circles for support during difficult times. Maintaining connections with accepting friends, family members, or partners provided a buffer against the negative effects of discrimination. This is consistent with studies showing that social support enhances mental health outcomes for LGBTIQ+ individuals by fostering a sense of belonging and reducing feelings of isolation (Bercea et al. 2023). Additionally, participants who sought solidarity with other LGBTIQ+ peers or accessed professional psychological support demonstrated relational resilience through community engagement and formal support systems.

The use of approach‐oriented coping strategies, such as seeking social support, engaging in meaningful activities, and defending oneself against discrimination, was associated with empowerment and resilience among participants. Furthermore, dreaming about the future and drawing inspiration from motivational stories or feminist activism reflect proactive strategies that foster hope and resilience. This aligns with international research that consistently shows that approach‐oriented strategies are linked to positive psychosocial adjustment and reduced mental health risks (e.g., Compas et al. 2001). For example, mindfulness practices have been found to mitigate the effects of stigma and improve mental health among LGBTIQ+ individuals (Chan and Leung 2021).

In contrast, some participants relied on avoidance‐oriented strategies such as moving away from the issue, tuning out phobic attitudes, and letting things slide. They also described a range of strategies which may reflect short‐term or maladaptive responses to stress (e.g., alcohol use, sleeping, self‐isolation). We acknowledge that, even though these behaviors can be understood as attempts to manage distress and may provide temporary relief from stressors, they are generally associated with poorer long‐term outcomes. Avoidance‐oriented coping has been linked to increased psychological distress and a heightened risk of mental health disorders among LGBTIQ+ individuals (Lehavot 2012; Sandfort et al. 2009; Seelman et al. 2017). For instance, ignoring stressors or isolating oneself can exacerbate feelings of loneliness and hinder emotional processing. Participants who avoided addressing discrimination directly or refrained from coming out due to fear of societal rejection exemplify the protective but ultimately limiting nature of avoidance coping. While these strategies may shield individuals from immediate harm (ERA LGBTI 2014), they can reinforce internalized stigma, reduce opportunities for personal growth and social connection (e.g., DiPlacido 1998; Frost and Meyer 2023; Hatzenbuehler 2009).

### 4.3 Implications and Future Directions

These findings collectively highlight the importance of systemic reforms to ensure equitable access to education, employment, and healthcare, as well as measures to promote the mental well‐being of LGBTIQ+ community and other marginalized groups in Turkey, and potentially in other Global Majority countries facing similar issues.

The study reveals pervasive educational discrimination, including peer bullying and systemic exclusion, particularly for those openly expressing their SOGI. Evidence from the Global North suggests that addressing these challenges requires implementing whole‐school approaches that foster inclusivity, professional development for educators, inclusive curricula, and enumerated anti‐discrimination policies (Day et al. 2016; Russell et al. 2021). For example, inclusive curricula that represent diverse SOGI experiences have been shown to promote understanding and reduce prejudice (Harris et al. 2021). In Turkey, however, research on LGBTIQ+ inclusivity in education is scarce (Coskun 2024), necessitating targeted studies to guide interventions.

Workplace discrimination remains a critical issue, with many LGBTIQ+ individuals in Turkey experiencing marginalization or being forced to conceal their identities. International evidence supports the effectiveness of anti‐bias training for employees and managers in fostering inclusive workplaces (Carter et al. 2020). In addition, creating explicit workplace protections for SOGI is essential. For example, legislative reforms in other countries have demonstrated that explicit anti‐discrimination laws can reduce workplace bias and improve job satisfaction among LGBTIQ+ employees. In Turkey, where such protections are absent (Özdemir and Açıkgöz 2024), advocacy for legal reforms is critical.

Ensuring equitable access to healthcare for LGBTIQ+ individuals in Turkey demands systemic reforms. These include legal protections against discrimination, mandatory anti‐bias training for healthcare professionals, and the integration of inclusive practices into medical curricula. Such measures would not only safeguard the health rights of LGBTIQ+ individuals but also contribute to a more inclusive and equitable healthcare system.

Findings also reveal significant barriers to equal legal protection and citizenship rights for LGBTIQ+ individuals in Bursa. Many participants reported experiencing discrimination in legal proceedings or when accessing municipal services, consistent with broader patterns of systemic exclusion documented in Turkey. The study further highlights the prevalence of hate speech in the media and press. Given the media's influence on public attitudes, this normalization of hate speech not only marginalizes LGBTIQ+ individuals but also reinforces discriminatory societal structures. The Turkish government has exacerbated this issue by actively promoting anti‐LGBTIQ+ rhetoric through public service announcements and media censorship, such as banning films and online content featuring LGBTIQ+ themes (Yildirim 2024). Strengthening legal mechanisms to prevent hate speech in the media is therefore essential. Where such mechanisms exist but remain inactive, enforcement must be prioritized to curtail the harmful impact of discriminatory narratives.

This study also emphasizes critical practice and policy implications for addressing the mental health disparities experienced by LGBTIQ+ individuals due to discrimination. These implications focus on fostering resilience, implementing targeted interventions, and creating inclusive environments that mitigate the adverse effects of minority stress. Interventions aimed at enhancing self‐awareness, self‐agency, and approach‐oriented coping strategies, such as mindfulness practices and activism, can empower LGBTIQ+ individuals to navigate adversity effectively. Research supports that mindfulness‐based interventions reduce stigma‐related stress and improve mental health outcomes (Chan and Leung 2021). In addition, fostering relational resilience through social support networks is essential. Studies show that strong social connections buffer against discrimination's negative effects by reducing isolation and promoting a sense of belonging (Bercea et al. 2023). Programs that facilitate peer support groups or community engagement for LGBTIQ+ individuals could amplify these benefits. This is especially important for LGBTIQ+ community in Bursa, where options for LGBTIQ+ individuals to socialize are quite limited.

Furthermore, mental health practitioners should prioritize interventions that encourage emotional processing and proactive problem‐solving. Avoidance‐oriented coping strategies, such as substance use or disengagement, could provide temporary relief but are associated with long‐term psychological distress (Lehavot 2012). Cognitive‐behavioral therapy and other evidence‐based approaches can help individuals shift from avoidance to approach‐oriented strategies, fostering better mental health outcomes (Pachankis et al. 2023). Given the elevated rates of depression, anxiety, and suicidal ideation among LGBTIQ+ populations (e.g., Basar and Oz 2016; Ordek 2014; Watson and Tatnell 2022), mental health services must be tailored to address their unique needs. Training healthcare providers in culturally competent care is crucial to reducing barriers to access and ensuring equitable treatment. Programs like PRIDE have demonstrated success in enhancing provider competency and empathy, which could be adapted for use in diverse cultural contexts (Matsutaka et al. 2024).

It is important to emphasize that while strategies aimed at resilience‐building and individual coping can provide critical support for LGBTIQ+ individuals, they do not address the root causes of the challenges these populations face. These efforts, though valuable, primarily focus on mitigating the effects of discrimination rather than eliminating its sources. In the long term, achieving meaningful and sustainable improvements in the mental well‐being of LGBTIQ+ individuals requires systemic change. This includes formulating and implementing comprehensive policies designed to prevent prejudice, discrimination, and social inequalities at both societal and institutional levels. Structural interventions are essential to dismantle the systemic barriers that perpetuate marginalization. Anti‐discrimination laws explicitly protecting SOGI are a foundational step toward fostering equality. In addition, public education campaigns aimed at reducing stigma and promoting acceptance can help shift cultural attitudes over time, creating an environment where LGBTIQ+ individuals feel valued and supported.

### 4.4 Strengths and Limitations

While evaluating this study and its findings, the methodological strengths and limitations should also be considered. One of the strongest features of this study is the adoption of the co‐production approach. The members and volunteers of Özgür Renkler Derneği [Free Colours Association], who are also LGBTIQ+ rights activists, designed, conducted, and reported this study together with an academic researcher (i.e., the first author), who is also a member of the association. They were supported by two additional academic researchers, who were considered allies and experts. Hence, co‐production established the ground for the researchers with different perspectives and experiences to contribute to the design, implementation, and interpretation of this research. Another strength of the research is utilizing a mixed‐method design, combining quantitative and qualitative techniques. The mixed‐method design is advantageous since it features the stronger sides of both techniques, compensates the weaker ones, provides a holistic understanding, and presents richer, more detailed, and more comprehensive explanations regarding the phenomena (Johnson and Onwuegbuzie 2004).

In regard to its limitations, this is a cross‐sectional study, meaning that the data were collected from the participants at a single time‐point. Therefore, the study reports associations between study variables, and the term *predictor* was used only in a statistical sense; the results should not be interpreted as evidence of causality. Using longitudinal methods in future research would provide more conclusive information regarding the cause‐and‐effect relationship between variables.

## 5 Conclusion

When social justice and equality do not exist and are not taken into consideration, the psychological well‐being and mental health of individuals get poor (e.g., Calma 2009). Discrimination inhibits individuals' participation in life and might cause irrecoverable physical, social, and emotional wounds. Given all these, this study emphasizes the importance and necessity of developing and expanding empowering, protective, and preventive mechanisms and services (e.g., accessible psychosocial and judicial support mechanisms), while also fostering resilience and promoting approach‐oriented coping strategies among LGBTIQ+ individuals. It also informs strategies and policies to be developed for LGBTIQ+ people based on their experiences and needs. Advocacy works for LGBTIQ+ individuals to benefit from equal rights should be carried out and followed by local governments, non‐governmental organizations, and institutions conducting human rights‐focused work.

## Ethics Statement

The study was conducted in accordance with the ethical standards of the American Psychological Association and was approved by the Social Sciences Research Ethical Committee at Koc University, Turkey.

## Consent

Informed consent was obtained from all participants included in the study. No identifying information or case studies are included in this article. All participants have consented to the publication of the research findings.

## Conflicts of Interest

The authors declare no conflicts of interest.

## Supporting information

Supporting Materials R2.

## Data Availability

The data that support the findings of this study are available on request from the corresponding author. The data are not publicly available due to privacy or ethical restrictions.
