# Supplementary material for: “I Am Very Happy That We Are Such Beautiful People”: Lived Experiences, Perceived Discrimination, and Mental Health in an LGBTIQ+ Community in Turkey
Source: J Community Psychol. 2026 Feb 6;54(2):e70087. doi: 10.1002/jcop.70087 (PMC12880800; doi:10.1002/jcop.70087)
Supplement: Supplementary file 1 — Supporting Materials R2. [file JCOP-54-0-s001.docx]

**Supplementary Materials**

**Alternative to Figure 1**

*Percentage of Participants Reporting Perceived Individual Discrimination.*

|  | Never (%) | Rarely (%) | Sometimes (%) | Often (%) | Always (%) |
| --- | --- | --- | --- | --- | --- |
| Feeling unaccepted in Turkey | 4.3 | 7.2 | 14.5 | 23.2 | 50.7 |
| Feeling that people/society are against oneself | 4.3 | 8.7 | 15.9 | 29 | 42 |
| Being insulted | 14.5 | 14.5 | 26.1 | 15.9 | 29 |
| Exposure to physical violence | 55.1 | 18.8 | 10.1 | 4.3 | 11.6 |
| Exclusion | 17.6 | 36.8 | 14.7 | 19.1 | 11.8 |
| Discrimination while looking for a job | 49.2 | 6.3 | 14.3 | 17.5 | 12.7 |
| Discrimination while looking for a house | 46.8 | 9.7 | 16.1 | 11.3 | 16.1 |
| Discrimination at school | 19.7 | 13.6 | 13.6 | 21.2 | 31.8 |
| Discrimination at workplace | 41.3 | 14.3 | 14.3 | 19 | 11.1 |
| Discrimination on the street | 25 | 20.3 | 18.8 | 20.3 | 15.6 |
| Discrimination while shopping | 37.5 | 21.9 | 14.1 | 14.1 | 12.5 |
| Discrimination at restaurant, café, pub, etc. | 43.8 | 20.3 | 14.1 | 9.4 | 12.5 |
| Discrimination while getting service from the private sector | 50.8 | 20.6 | 12.7 | 4.8 | 11.1 |
| Discrimination at healthcare institutions | 43.8 | 15.6 | 18.8 | 10.9 | 10.9 |
| Discrimination while getting service in the field of law | 65.6 | 12.5 | 10.9 | 1.6 | 9.4 |
| Discrimination while getting service from the municipality | 69.3 | 8.1 | 9.7 | 3.2 | 9.7 |
| Discrimination on media and press | 37.1 | 12.9 | 14.5 | 12.9 | 22.6 |
| Discrimination on social media | 15.6 | 18.8 | 21.9 | 17.2 | 26.6 |
| Discrimination on dating websites and apps | 34.4 | 12.5 | 21.9 | 10.9 | 20.3 |

**Alternative to Figure 2**

Percentage of Participants Reporting Perceived Group Discrimination

|  | Never (%) | Rarely (%) | Sometimes (%) | Often (%) | Always (%) |
| --- | --- | --- | --- | --- | --- |
| Discrimination while looking for a job | 0 | 0 | 10.9 | 35.9 | 53.1 |
| Discrimination while looking for a house | 0 | 4.5 | 9.1 | 31.8 | 54.5 |
| Discrimination at school | 0 | 3 | 9 | 29.9 | 58.2 |
| Discrimination at workplace | 0 | 1.5 | 10.8 | 35.4 | 52.3 |
| Discrimination on the street | 0 | 3.1 | 7.7 | 35.4 | 53.8 |
| Discrimination while shopping | 1.5 | 7.7 | 13.8 | 36.9 | 40 |
| Discrimination at restaurant, café, pub, etc. | 3.1 | 10.8 | 20 | 29.2 | 36.9 |
| Discrimination while getting service from the private sector | 7.7 | 7.7 | 26.2 | 26.2 | 32.3 |
| Discrimination at healthcare institutions | 3.1 | 7.7 | 13.8 | 32.3 | 43.1 |
| Discrimination while getting service in the field of law | 4.7 | 10.9 | 17.2 | 34.4 | 32.8 |
| Discrimination while getting service from the municipality | 4.6 | 9.2 | 15.4 | 33.8 | 36.9 |
| Discrimination on media and press | 3.1 | 3.1 | 10.8 | 23.1 | 60 |
| Discrimination on social media | 1.5 | 0 | 7.7 | 29.2 | 61.5 |
| Discrimination on dating websites and apps | 1.8 | 27.7 | 24.6 | 35.4 | 10.5 |
